# All-Silicon Polarization-Insensitive Metamaterial Absorber in the Terahertz Range

**DOI:** 10.3390/ma17092098

**Published:** 2024-04-29

**Authors:** Zongcheng Xu, Yujie Li, Bin Han, Yue Wang, Quan Yuan, Yanan Li, Weiyan He, Junhua Hao, Liang Wu, Jianquan Yao

**Affiliations:** 1 Department of Physics, Tianjin Renai College, Tianjin 301636, China; liyujie_111@126.com (Y.L.); nkhanbin6@126.com (B.H.); yuewang_1024@tju.edu.cn (Y.W.); ewanyuan@tju.edu.cn (Q.Y.); lynan@163.com (Y.L.); heweiyan@aliyun.com (W.H.); nkhjh@mail.nankai.edu.cn (J.H.); 2 Key Laboratory of Opto-Electronics Information Science and Technology, Ministry of Education, Institute of Laser and Opto-Electronics, Tianjin University, Tianjin 300072, China; jqyao@tju.edu.cn

**Keywords:** terahertz, all-dielectric, broadband absorption, diffraction

## Abstract

All-silicon terahertz absorbers have attracted considerable interest. We present a design and numerical study of an all-silicon polarization-insensitive terahertz metamaterial absorber. The meta-atoms of the metamaterial absorber are square silicon rings which can be viewed as gratings. By properly optimizing the structure of the meta-atom, we achieve a broadband absorptivity that is above 90% ranging from 0.77 THz to 2.53 THz, with a relative bandwidth of 106.7%. Impedance matching reduces the reflection of the terahertz waves and the (0, ±1)-order diffraction induce the strong absorption. The absorption of this absorber is insensitive to the polarization of the terahertz wave and has a large incident angle tolerance of up to 60 degrees. The all-silicon metamaterial absorber proposed here provides an effective way to obtain broadband absorption in the terahertz regime. Metamaterial absorbers have outstanding applications in terahertz communication and imaging.

## 1. Introduction

Terahertz (THz) waves with frequencies ranging from 0.1 THz to 10 THz are located in the microwave and the infrared wave of the electromagnetic spectrum. Due to the relative scarcity of substances in nature that have a strong electromagnetic response to THz waves, high-performance THz functional devices have long been the bottleneck that limits the application of terahertz technology. Metamaterials have opened a new idea and provided a new means for the solution of this problem [1,2,3,4,5,6]. In these functional devices, metamaterial absorbers (MAs) have shown great applications in military stealth, thermal imaging, photovoltaic materials, detectors, and so on [7,8,9,10]. The first concept of metamaterial absorber (MA) was demonstrated by Landy et al. [11]. From then on, single-band, dual-band, triple-band, quad-band, penta-band, six-band, and hepta-band MAs have been proposed and investigated widely [12,13,14,15,16,17,18]. These MAs can be called single-band or multi-band MAs. Broadband THz MAs have attracted intense attention and a great deal of interest because of their urgent needs in THz shielding, imaging, polarization conversion, and related fields [19,20,21,22,23]. Once the structure of the metamaterial absorber is determined, the absorption situation is difficult to change. Kumar et al. demonstrates active tuning of metasurface resonances by photoinduced pumping power [24]. Ma et al. proposed a THz metamaterial absorber based on vanadium dioxide and graphene and achieved large broadband absorption and perfect dual narrowband absorption. The absorption can be switched from broadband to dual narrowband [25]. Because of its excellent properties, graphene is one of the ideal choices for the extraction and tuning of metamaterial absorbers. Li et al. proposed an active tunable terahertz absorber based on single-layer graphene to realize broadband absorption [26]. Kumar D et al. studied a terahertz graphene metasurface exhibiting dynamic slow light behavior and proposed a new method to enhance bandwidth [27,28]. Zheng et al. proposed a terahertz absorber based on monolayer graphene, which can realize broadband absorption in the terahertz band [29]. Ri et al. proposed a dual-broadband THz metamaterial absorber based on a single asymmetric resonator [30]. However, up to now, how to realize broadband MAs still remains a challenge. In order to expand the bandwidth, the main method is to incorporate multiple resonant modes on a plane [31,32,33]. These MAs usually consist of three layers (metal-insulator-metal), where the top and the bottom layers are metal. It can achieve the impedance matching by coupling the top layer and the bottom layer. Recently, all-dielectric MAs have been proposed to realize perfect absorption [34,35,36,37,38]. In addition, there has been considerable interest in designing MAs based on silicon [39,40,41]. Li and Coopers proposed a THz absorber based on high-resistance silicon and also achieved absorption greater than 90% in the range of 0.58 to 1.92 THz [42]. Subwavelength silicon nanocylinders can be designed to possess dielectric interference coatings and yield almost zero reflectance over the entire spectral range from the ultraviolet to the near-infrared [43]. One-dimensional semiconductor silicon nanowires can significantly enhance the absorption of sunlight [44]. A simple structure, dumbbell-shaped doped-silicon grating arrays, is proposed to demonstrate broadband absorption [45]. The property of absorption of this absorber is realized mainly based on the mechanisms of the anti-reflection effect and grating diffractions. Zhao et al. presented an all-silicon broadband MA with an absorbance bandwidth of 0.9 THz [46]. The technology of semiconductor silicon is easy to fabricate and low-cost. In a word, all-silicon metamaterials have great potential in the development of the THz MAs.

In this letter, we present a novel design and numerical investigation of an all-silicon polarization-insensitive terahertz (THz) MA based on undoped silicon. By optimizing the meta-atom structure appropriately, we achieve an absorptivity above 90%, ranging from 0.77 THz to 2.53 THz when the conductivity of semiconductor silicon is set as 150 S/m. The entire thickness of the all-dielectric THz MA is only about 550 μm. The square ring structure on the upper layer of the MA acts as an anti-reflection layer, and together with the base forms the broadband absorber. To understand the physical mechanism of the absorption properties, we simulate the electric field and the power loss density of the THz MA. The diffraction plays a crucial role.

## 2. Structural Design and Simulation

**Figure 1:**
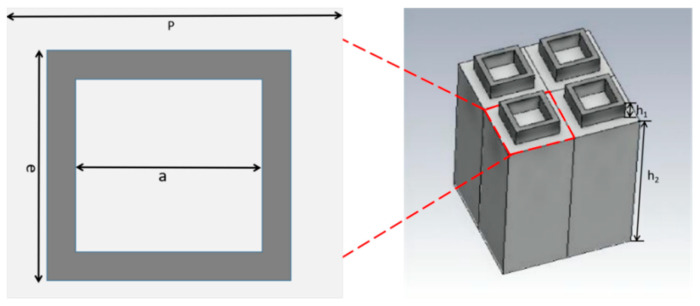
Structure schematic of the proposed all-dielectric THz MA. The top view of the meta-atom on the left and the side view of the structure on the right. The all-silicon THz MA shows the following dimensions: *p* = 120 μm, a = 60 μm, e =80 μm. The thickness of the square silicon ring is h_1_ = 50 μm, while h_2_ = 500 μm is the thickness of the silicon substrate. The total thickness of all-silicon THz MA we utilize in simulation is 550 μm.

Figure 1 shows a schematic diagram of the proposed all-dielectric MA. The substrate is undoped semiconductor silicon, with a thickness of 500 μm, which can block the transmission of THz waves over the entire investigated frequency range when the conductivity of semiconductor silicon is set as 150 S/m. The structure of the unit cell, which we call meta-atom, is designed as a square silicon ring. The period of the MA is 120 μm, while the thickness of the square silicon ring is 50 μm. The length of the square silicon ring is 80 μm with a width of 10 μm. We chose the structure of square rings because the structure of a square ring can lead to resonance absorption. In our previous works, we have numerically studied the resonance characteristics of MA by using square rings [41].

**Figure 2:**
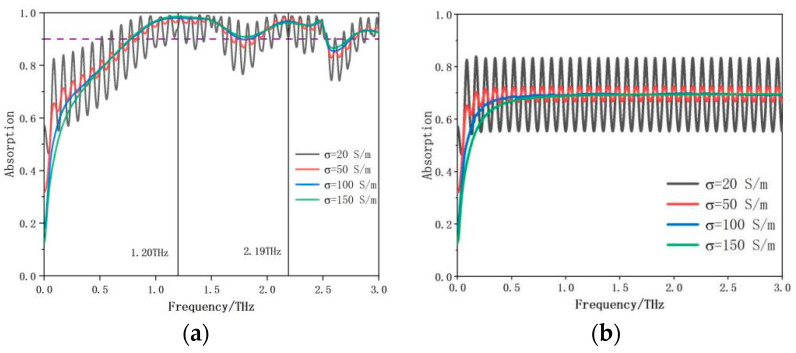
(**a**) Simulated terahertz absorption spectrum of the proposed MA for the different values of silicon conductivity at 20 S/m, 50 S/m, 100 S/m and 150 S/m, respectively. (**b**) Simulated terahertz absorption spectrum with only silicon wafer for the different values of silicon conductivity at 20 S/m, 50 S/m, 100 S/m and 150 S/m, respectively.

The commercial software program CST Microwave Studio 2019 was used to simulate and optimize the all-dielectric THz absorber structure. A normal transverse magnetic (TM) wave is used in the simulation so that the electric and magnetic fields are parallel to the x and y axes, respectively. An open boundary condition is employed along the z-direction. A total of 170,035 tetrahedron mesh cells are generated during the numerical process. It takes about three hours to run a simulation. The absorption can be calculated by A(f)=1−R(f)−T(f), where $R(f)={S_{11}}^{2}$ represents the reflection and $T(f)={S_{21}}^{2}$ represents the transmission of the all-dielectric THz absorber in our simulation. We also simulate the reflection and transmission of only silicon wafer with different conductivities. The absorption of the silicon wafer is shown in Figure 2. The conductivity of semiconductor silicon can be easily changed by applying pump laser power. We achieve a broadband absorption when the conductivity of the silicon is assumed to be 150 S/m in the simulations.

## 3. Results and Discussion

Figure 2a shows the simulated absorption spectrum of the proposed all-silicon MA for the different values of silicon conductivity at 20 S/m, 50 S/m, 100 S/m and 150 S/m, respectively. When the value of the conductivity is low, the oscillation range of the absorption curve is large. The absorption rate above 90% ranges from 0.77 THz to 2.53 THz when the conductivity of silicon is set at 150 S/m. After calculation, the relative bandwidth of the MA is 106.7%. We also calculate the absorption of separate silicon wafer with different conductivity, as shown in Figure 2b. It can be clearly seen that the oscillation range is large when the value of the conductivity of silicon is 20 S/m. As the value of the conductivity of silicon increases to 100 S/m and 150 S/m, the absorption rate is stable at 70%. Therefore, the substrate of the semiconductor silicon plays a key role for the proposed MA. The square ring structure on the upper layer of the MA acts as the anti-reflection layer, and together with the base make up the broadband MA. The proposed all-silicon THz MA has two distinct resonance absorption points at frequencies 1.20 THz and 2.19 THz.

**Figure 3:**
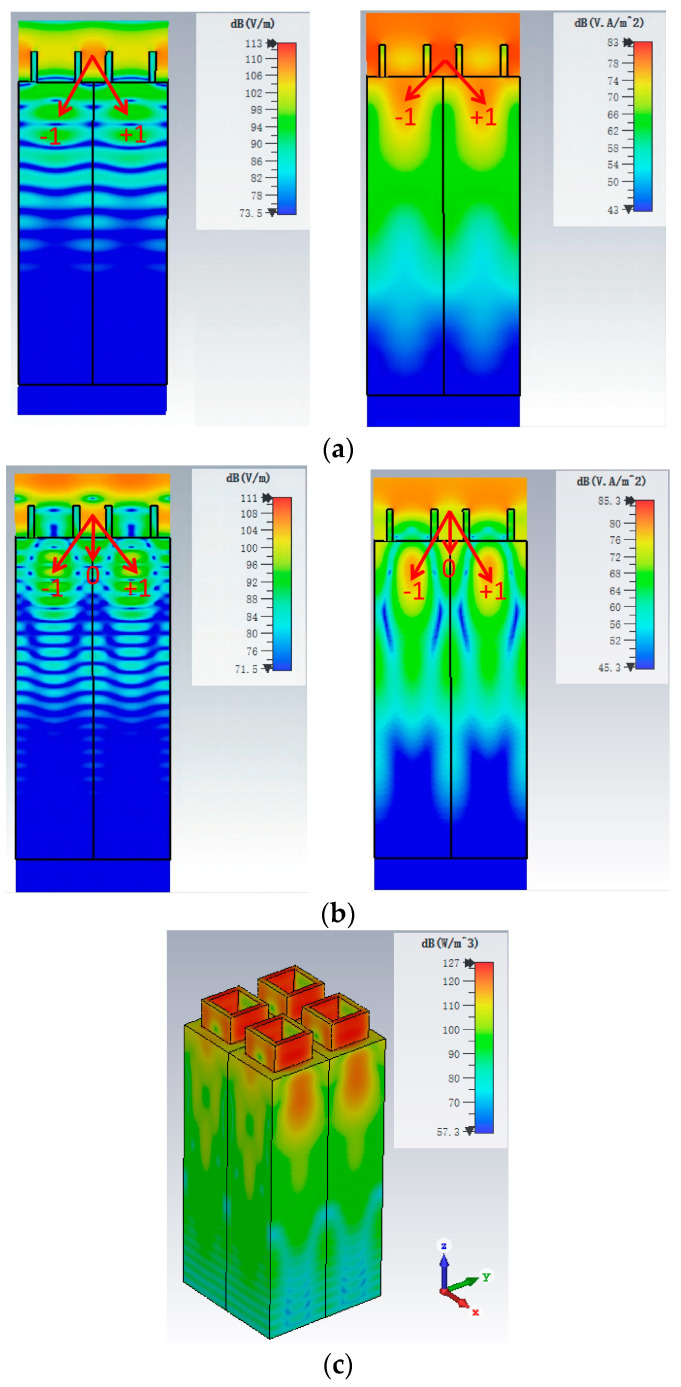
Electric field and the power loss density of the proposed THz MA at (**a**) 1.20 THz and (**b**) 2.19 THz resonance modes when the conductivity is 150 S/m. These figures are cross-sections on y-z plane. (**c**) The power loss density per unit volume of the proposed THz MA at 2.19 THz when the conductivity is 150 S/m.

To understand the physical mechanism of the absorption properties, we simulated the electric field and the power loss density of the proposed MA at 1.20 THz and 2.19 THz, as shown in Figure 3. When THz wave is incident vertically on the MA, the (−1, +1)-order diffraction plays an important role at the low frequency 1.20 THz, as shown in Figure 3a. The top layer of the all-silicon MA is a single-layer square silicon array, which can be viewed as diffraction grating. Grating is a device on which there are a very large number of parallel, identical, close-spaced slits. The maxima of intensity (constructive interference) can be deduced by the diffraction grating equation [36]:

ndsinθ=kλ

where n is the refractive index of the undoped silicon, d is spacing between adjacent slits, θ is angular separation between the order of maxima, k (k=0, ±1, ±2, ±3,⋯⋯) is the order of maxima, and λ is the wavelength of the THz wave in vacuum. When THz wave (1.20 THz) passes through the slits (spacing between square rings) of the diffraction grating, there is constructive interference at the first order maxima. It can be clearly seen from the electric field and the power loss density. In our proposed THz MA, we can calculate the diffraction grating with a slit spacing of 120 µm from Figure 1. At the first order maxima, we can also calculate the angle of diffraction is about 37.1° according to the equation $\theta =\mathrm{arc}\;\sin(\frac{\lambda }{np})$ at 1.20 THz. In the calculation, the spacing between adjacent slits (d) is equal to the period (*p* = 120 µm) of the meta-atom and the refractive index of the undoped silicon (n) is about 3.45. When f=1.20 THz, its corresponding wavelength is 250 µm, which is greater than the spacing between adjacent slits. It can produce significant diffraction for the proposed all-dielectric THz MA. When f=2.19 THz, the (0, ±1)-order diffraction plays an important role for the absorption, as shown in Figure 3b. When a THz wave passes through the slits of the diffraction grating, the path difference at the zeroth order maximum is zero. At the first order maxima, it can produce constructive interference to absorb the incident THz wave. The distribution of electric field and power loss density shows that dominant absorption component is concentrated between the upper silicon rings and the top part of the substrate of silicon both of the two resonant absorption frequencies. Figure 3c shows the power loss density per unit volume of the proposed THz MA at 2.19 THz when the conductivity is 150 S/m. It is also clear that the terahertz wave energy is mainly concentrated in the upper antireflection layer and the upper part of the substrate.

**Figure 4:**
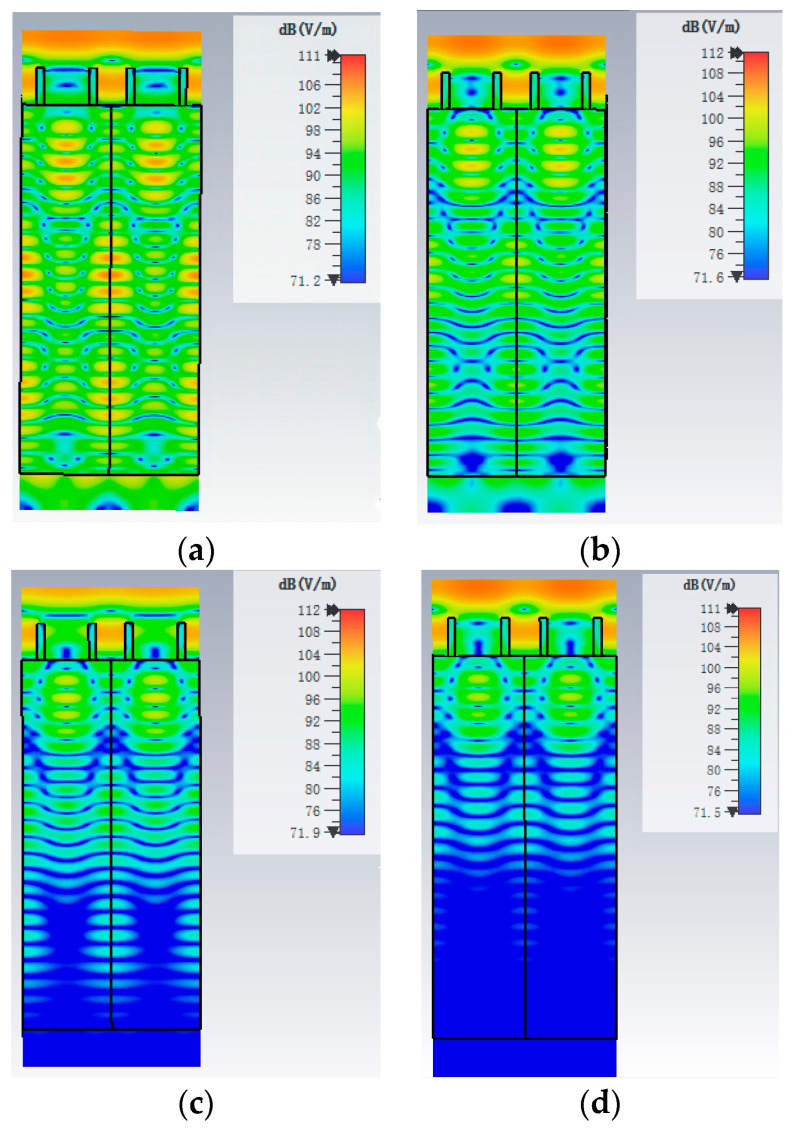
Electric field distributions of the broadband MA at frequency 2.19 THz with different conductivities (**a**) 20 S/m (**b**) 50 S/m (**c**) 100 S/m (**d**) 150 S/m, respectively. These figures are cross-sections on y-z plane.

The conductivity of semiconductor silicon exhibits a dependence on the intensity of external optical excitation, resulting in a significant increase in photogenerated carriers. Therefore, we simulate the electric field distributions of the broadband MA at frequency 2.19 THz with four different conductivities: 20 S/m, 50 S/m, 100 S/m, and 150 S/m, as shown in Figure 4. When the conductivity of silicon is 20 S/m, the distribution of the electric field is concentrated on the upper silicon ring structure and the entire silicon substrate, as shown in Figure 4a. The (−1, +1)-order diffraction is mainly concentrated on the top of the silicon substrate. The zeroth order diffraction is transmitted to the entire silicon substrate. When the conductivity of silicon is 50 S/m, the phenomenon of absorption is similar to that of the conductivity of 20 S/m. However, it can be seen from Figure 4b that the strength of the electric field decreases with the distance transmitted in the substrate. When the conductivity of silicon is 100 S/m, the strength of the electric field decreases further with the increase in distance, as shown in Figure 4c. When the conductivity of silicon is 150 S/m, fewer electric fields enter the lower part of the substrate, as can be seen from Figure 4d. This indicates that the carrier densities in silicon decrease along the propagation direction. Therefore, we cannot assume the carrier concentration to be uniform in the model of semiconductor undoped silicon. It can also be interpreted from the Beer−Lambert law that the influence of external optical excitation on the lower part of the semiconductor is attenuated. The absorption for terahertz waves in the lower part is weakened. This implies that selecting a thinner silicon substrate could be considered for designing a broadband THz MA.

**Figure 5:**
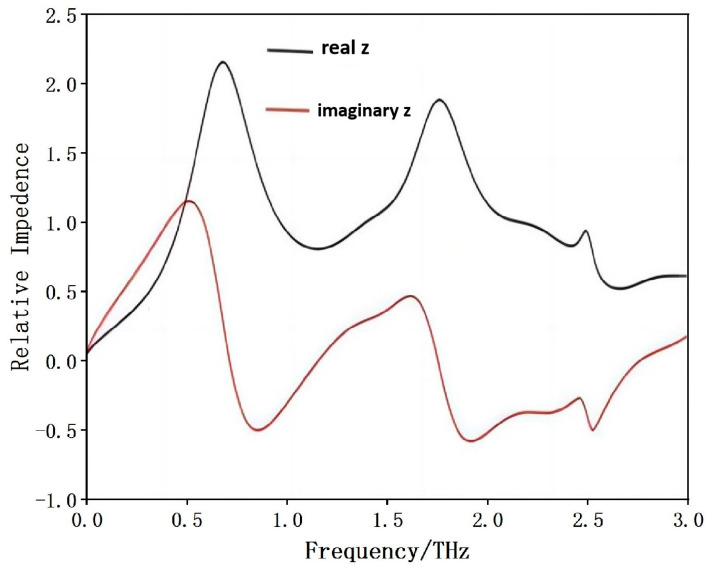
The relative impedance of the broadband metamaterial absorber at normal incidence.

Silicon nanostructures exhibit powerful optical and nonlinear optical properties, which can display enhanced localized electric fields at resonance frequencies [47]. The asymmetric device exhibits a sharp narrow-band resonance, while the symmetric device can widen the absorption bandwidth [48]. The wavelength range of THz wave is 0.03 mm~3 mm, which is greater than the structural size of the periodic unit. Therefore, the proposed broadband THz MA can be considered as uniform medium. We calculate the relative impedance of the all-silicon THz MA to further explain and investigate the absorption performance. The relative impedance can be calculated as follows:

$$z=\pm \sqrt{\frac{{\left(1+S_{11}\right)}^{2}-{S_{21}}^{2}}{{\left(1-S_{11}\right)}^{2}-{S_{21}}^{2}}}$$

where *S*_11_ is complex reflection coefficient, and *S*_21_ is complex transmission coefficient. The impedance of the free space is one, which suggests that the relative impedance must be one in order to match the free space and the MA. In other words, the real value of the relative impedance must require near one and the imaginary part of the relative impedance appear to near zero. The relative impedance of the all-silicon is shown in Figure 5. We can also clearly see that the real part and the imaginary part of the relative impedance is nearly one and zero at 1.20 THz and 2.19 THz. This all-silicon MA is a wideband absorber from 0.77 THz to 2.53 THz with an absolute bandwidth of 1.76 THz. If the real part of the all-silicon THz MA is greater than or equal to 0.52 and less than or equal to 1.99, the absorptive efficiency of the MA can exceed 90%. As we can see from Figure 5, the relative impedance matches very well in the wide frequency range.

**Figure 6:**
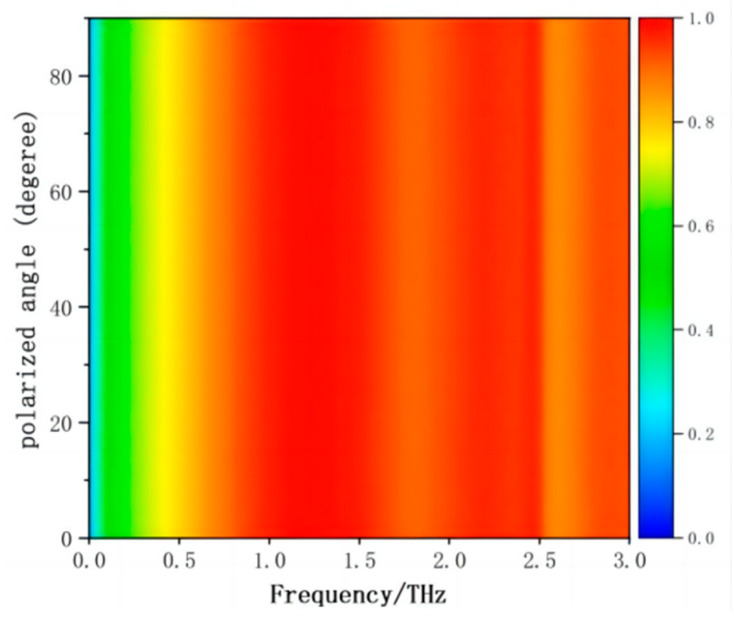
The simulated absorption spectrum of all-silicon MA. The terahertz waves are normally incident on the all-silicon THz absorber. The polarization angles were calculated from zero degrees to ninety degrees with the step of ten degrees.

**Figure 7:**
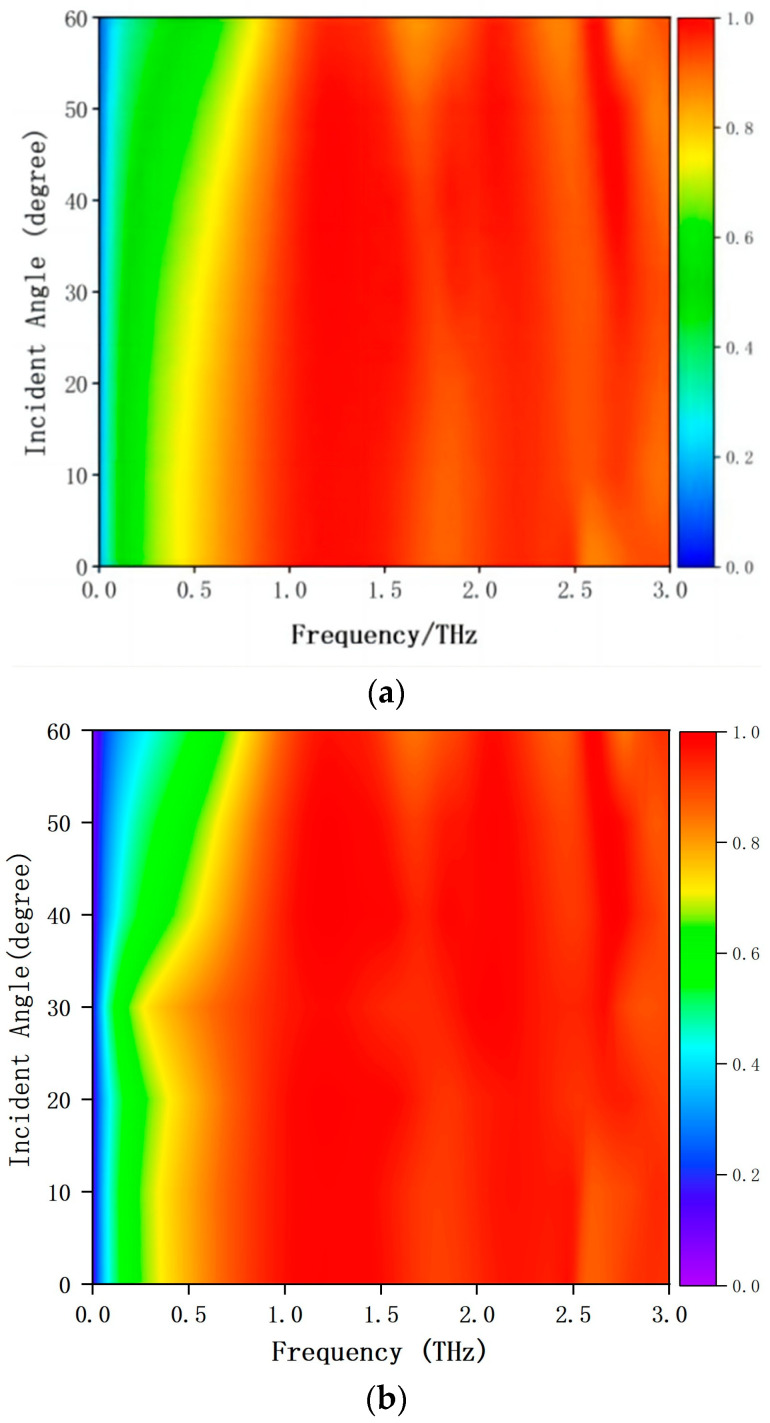
The simulated all-silicon broadband THz absorption spectrum. The oblique incidence was calculated from zero to sixty degrees for (**a**) TM polarization and (**b**) TE polarization with the step of ten degrees.

Polarization and wide angle incidence are very necessary for the practical application of THz MA. The proposed of all-silicon THz MA is insensitive to the polarization angle due to the four-fold rotational symmetry of the square silicon rings as shown in Figure 6. Here, we consider the THz radiation for TM wave and TE wave. Figure 7 shows the all-dielectric THz absorption spectrum under different incident angles step in 10 degrees. The absorption remains still greater than 90% in a wide frequency range with the incident angle shifts up to 60 degrees for TM and TE wave. However, the absorption strength is decreased in the low frequency range. It can be concluded that this all-silicon MA is sensitive to the incident angles from zero to sixty degrees. From the image, the absorption of TE wave is better than that of TM wave.

We list some key characteristics and features in Table 1 in order to compare our work with some previous works. We list five main aspects that are measured: band, absorption, relative bandwidth, relative bandwidth, meta-atoms, and thickness. It is obvious that the meta-atoms squared silicon rings are fairly simple compared to others. Compared with the three-layer absorber structure, the proposed absorber is realized by etching silicon and is relatively easy to design.

## 4. Conclusions

In summary, an all-silicon polarization-insensitive THz MA has been proposed. The meta-atom is designed as a square silicon ring and the substrate is also undoped semiconductor silicon. Therefore, we call this proposed absorber an all-silicon broadband MA. The meta-atoms of the MA can be viewed as gratings. Impedance matching reduces the reflection of the terahertz waves and the (0, ±1)-order diffractions induce the strong absorption. The absorptivity above 90% ranges from 0.77 THz to 2.53 THz with an absolute bandwidth of 1.76 THz. The proposed all-dielectric THz absorber has two distinct resonance absorption points at 1.20 THz and 2.19 THz, respectively. The (−1, +1)-order diffraction plays an important role at the low frequency, while the (0, ±1)-order diffraction plays an important role for the absorption at the higher frequency. The distribution of electric field and power loss density shows that the dominant absorption component is concentrated in the upper silicon ring and the top part of the silicon substrate at the two resonant absorption frequencies. According to the impedance matching principle, the relative impedance of the proposed THz MA matches very well in a wide frequency range. The proposed all-silicon THz absorber is insensitive to the polarization angle due to the four-fold rotational symmetry of the square silicon rings. This all-silicon MA is also sensitive to the incident angles from zero to sixty degrees. In the future we want to further optimize the structural design of the wideband metamaterial absorber and find other ways to improve the absorption bandwidth of the absorber, such as replacing the current material with a phase-changing material to enhance the bandwidth, and theoretically analyze the effects of near-field interactions and the coupling properties between the meta-atoms.

## Figures and Tables

**Table 1:** Comparison of the previous broadband metamaterial absorbers with the proposed metamaterial absorber.

Refs.	Absorption	Absorption Band (THz)	Relative Bandwidth	Meta-Atoms	Thickness (μm)
[3]	≥80%	0.473–1.407	97.8%	cross-shaped grooves	95.2
[25]	≥50%	1.24–2.85	79.0%	a rectangular-shaped resonator having an elongated slot	14 (middle layer)
[32]	≥90%	1.1–1.6	38.5%	a n-doped silicon membrane with elliptical holes	75
[33]	≥90%	0.67–1.78	90.6%	cross structures etched into a doped silicon substrate	265
[35]	≥90%	0.58–1.92	107.2%	ellipse pillar	500
[38]	≥95%	0.92–2.4	89.2%	two 90 degree crossed dumbbell-shaped doped-silicon grating	500
our work	≥90%	0.77–2.53	106.7%	square silicon rings	550

## Data Availability

Data are contained within the article.
